# Two Types of Contact Between Lipid Droplets and Mitochondria

**DOI:** 10.3389/fcell.2020.618322

**Published:** 2020-12-15

**Authors:** Liujuan Cui, Pingsheng Liu

**Affiliations:** ^1^National Laboratory of Biomacromolecules, CAS Center for Excellence in Biomacromolecules, Institute of Biophysics, Chinese Academy of Sciences, Beijing, China; ^2^University of Chinese Academy of Sciences, Beijing, China

**Keywords:** contact, lipid droplets, mitochondria, anchor, isolation, centrifugal force

## Abstract

Lipid droplets (LDs) and mitochondria are essential organelles involved in cellular lipid metabolism and energy homeostasis. Accumulated studies have revealed that the physical contact between these two organelles is important for their functions. Current understanding of the contact between cellular organelles is highly dynamic, fitting a “kiss-and-run” model. The same pattern of contact between LDs and mitochondria has been reported and several proteins are found to mediate this contact, such as perilipin1 (PLIN1) and PLIN5. Another format of the contact has also been found and termed anchoring. LD-anchored mitochondria (LDAM) are identified in oxidative tissues including brown adipose tissue (BAT), skeletal muscle, and heart muscle, and this anchoring between these two organelles is conserved from mouse to monkey. Moreover, this anchoring is generated during the brown/beige adipocyte differentiation. In this review, we will summarize previous studies on the interaction between LDs and mitochondria, categorize the types of the contacts into dynamic and stable/anchored, present their similarities and differences, discuss their potential distinct molecular mechanism, and finally propose a working hypothesis that may explain why and how cells use two patterns of contact between LDs and mitochondria.

## Introduction

The lipid droplet (LD) is a specialized cellular organelle with a neutral lipid core covered by a monolayer phospholipid membrane and associated proteins (Murphy, 2001; Tauchi-Sato et al., 2002; Fujimoto et al., 2008; Thiam et al., 2013). Starting with the discovery by Antony van Leeuwenhoek in 1674, LD biology has taken a sweeping 300 year-long developmental path to the modern era. Our understanding has accelerated in the last two decades, importantly marked by the discovery of marker proteins, development of methods for purification, and the application of omics studies (Ding et al., 2013; Roberts and Olzmann, 2020).

The LD is an ancient organelle with a new face and a recent recognition of its importance has attracted great attention in the fields of biology and life science. The newly discovered fact that LDs exist in some bacteria extends our view of the origin of membrane-bound organelles. The finding that LDs contain not only triacylglycerol (TAG), but also cholesterol ester, retinal ester, and monoalk(en)yl diacylglycerol in animal cells (Bartz et al., 2007a) suggests many potential roles for this organelle. In addition, the broad distribution of LDs from bacteria to humans implies its significance in all organisms (Murphy, 2012; Olzmann and Carvalho, 2019; Zhang and Liu, 2019).

The unique property of LDs in their sequestration of neutral lipids led to a perception of them merely as “a drop of oil,” negatively impacting scientific interest and slowing down its research. However, the description of their proteome and lipidome and their linkage to metabolic syndromes impacting human health provoked a renaissance in research interest and activity (Brasaemle et al., 2004; Liu et al., 2004; Bartz et al., 2007a). The LD differs from other membrane-bound organelles by containing (1) a hydrophobic core compared with an aqueous phase lumen for other membranous organelles, (2) a monolayer phospholipid membrane in contrast with the bilayer or double bilayer membrane for others, (3) a distinct protein complements termed LD resident proteins. With these differences, the LD is a unique cellular organelle. The functions of the LD are lipid storage, lipid transportation, lipid synthesis, and lipid hydrolysis, a suite of functions far beyond their image as a static oil reservoir. According to recent proteomic and other experimental lines of evidence, LD functions are also thought to include membrane trafficking, protein storage and degradation, signal transduction, detoxification, and nucleic acid handling. The multifunctional property of the LD endows the organelle with an irreplaceable position in almost all cellular activities.

The LD is integrated with other organelles in maintaining lipid homeostasis (Walther and Farese, 2012; Schuldiner and Bohnert, 2017), which is responsible for the ectopic storage of lipids that has been linked to many metabolic disorders (Greenberg et al., 2011; Shulman, 2014; Xu et al., 2018). LDs possess the ability for directed movement on the cytoskeleton, likely supporting the movement of neutral lipids within the cell and between organelles. Biochemical studies previously revealed the interaction between LD and early endosome (Liu et al., 2007), mitochondria (Pu et al., 2011), endoplasmic reticulum (ER) (Martin et al., 2005; Ozeki et al., 2005), and peroxisomes (Binns et al., 2006). More interactions between LDs and other organelles have been developed in the past decade (Olzmann and Carvalho, 2019). Therefore, LD-governed cellular lipid homeostasis is driven by lipid metabolism on the LD as well as the interaction between LD and other organelles. For example, TAG sequestered in the LD is converted to fatty acids (FAs) via lipolysis and lipophagy (Zechner et al., 2017; Ogasawara et al., 2020). The FAs in turn, are transported from LD to mitochondria, where they are oxidized as an energy source to produce ATP or heat, and provide building blocks for synthesis of biological molecules. Thus, the LD and mitochondrion form a functional organelle pair, managing cellular energy homeostasis.

**FIGURE 1:**
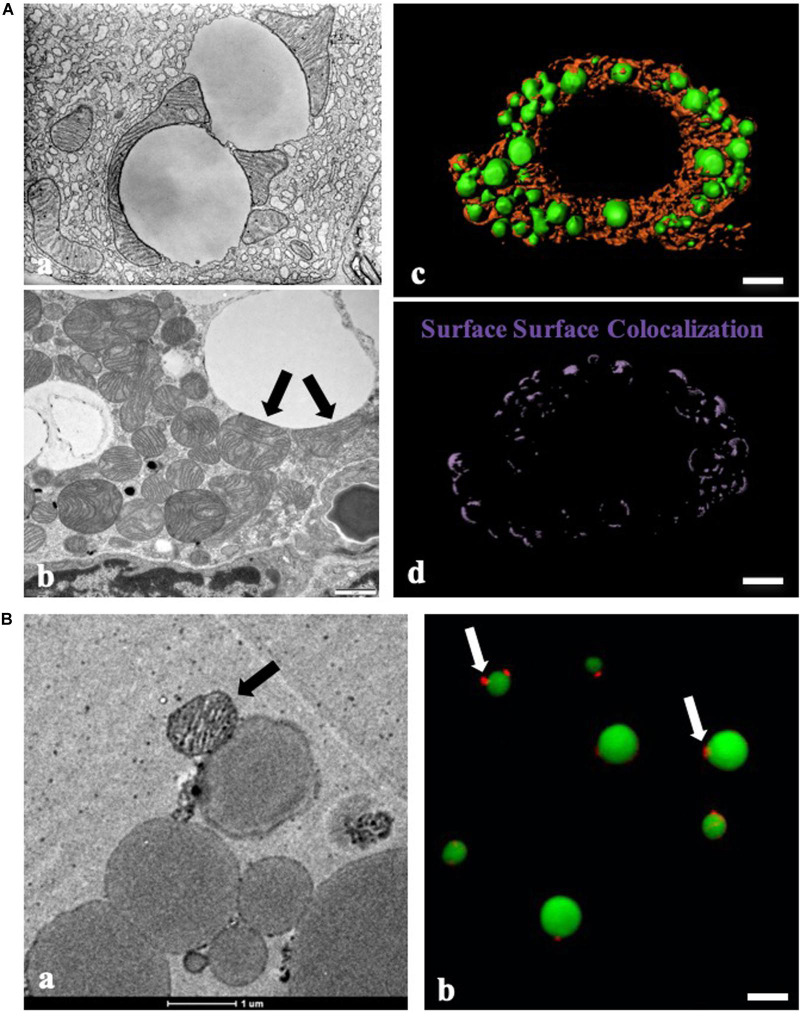
Contact between LDs and mitochondria by morphological studies. **(A)** Morphological study of contact between lipid droplets (LDs) and mitochondria. **(a)** Physical contact between LDs and mitochondria in guinea pig pancreas imaged with transmission electron microscopy (TEM) by George E. Palade (Cite from http://www.microscopy.info/Gallery/Details/58). **(b)** Physical contact between LDs and mitochondria in mouse BAT using TEM. Arrows point to the physical contact between LDs and mitochondria. **(c,d)** LDs and mitochondria in differentiated brown adipocytes (day 8) were stained with LipidTOX Green for LDs and MitoTracker Red for mitochondria, respectively. The image was analyzed using three-dimensional structured illumination microscopy (3D-SIM), and Imaris analysis was applied to detect surface-surface colocalization. Bar = 5 μm. **(B)** Morphologic analysis of isolated BAT LDs from mice housed at 23°C. **(a)** TEM of isolated BAT LDs, Bar = 1 μm. **(b)** LipidTOX Green staining for LDs and MitoTracker Red staining for mitochondria in isolated BAT LDs. Bar = 5 μm. Arrows indicate LD-anchored mitochondrion (LDAM) in isolated BAT LDs (cite from Cui et al., 2019).

Mitochondria are the site of β-oxidation, converting hydrophobic substrates into usable cellular energy and reducing potential. The fuel for this reaction is stored in the hydrophobic interior of LDs, necessitating a mechanism for the transfer of FAs between these two organelles. Free transfer of FA (or coenzyme A ligated FA) is restricted by the hydrophobic character of the molecules. In fact, the accumulation of FA or acyl-CoA under certain stress conditions leads to cellular toxicity (lipotoxicity). To facilitate the transfer, cells developed a specific interaction between these two organelles. An understanding of this interaction was initiated by the visualization of the physical contact between LDs and mitochondria. The best images depicting this interaction were electron micrographs taken by George Palade, in which the physical contact was clearly shown between LDs and mitochondria in guinea pig pancreas (Figure 1Aa). Since then, the development of high resolution light microscopy techniques and the availability of dyes and marker proteins for both LDs and mitochondria have greatly facilitated the visualization of the association of the two organelles.

Despite early, clear visual evidence of the contact, it has been a long journey toward a biochemical understanding of the interaction. Investigations were hampered by underdeveloped techniques for biochemical isolation and a lack of molecular tools. The discovery of LD marker proteins perilipin (PLIN1) and adipocyte differentiation-related protein (ADRP/PLIN2) in the early 1990s (Greenberg et al., 1991; Jiang and Serrero, 1992; Brasaemle et al., 1997) permitted the development of purification techniques and an evaluation of the purity of isolated organelle (Wu et al., 2000; Ding et al., 2013). These techniques coupled with the blossoming of proteomics opened a window into the functions, interactions, and underlying molecular machinery governing LD dynamics. These advances permitted the discovery that other organelles, like ER and mitochondria, co-isolate with LDs. Morphological studies provided further evidence for inter-organelle contact while proteomic results identified likely protein candidates regulating the contact. Interestingly, it has been observed that some organelles which are co-purified with LDs when isolated with low speed centrifugation can be separated from the LDs in higher centrifugal force, while other remain firmly associated (Yu et al., 2015; Benador et al., 2018; Cui et al., 2019; Freyre et al., 2019). This distinction probably reflects underlying functional relationships. For example, LDs isolated from heart muscle (Li et al., 2016), skeletal muscle (Zhang et al., 2011), and brown adipocytes (Yu et al., 2015; Cui et al., 2019) always contain many mitochondrial proteins that were once considered contamination. On the other side of the co-isolation, some LD-associated proteins have also been identified on mitochondria, such as PLIN5 and PLIN3 (Bosma et al., 2012; Ramos et al., 2014). However, these proteins cannot be removed by ultracentrifugation. With morphological and biochemical evidence, these proteins reflect a stable contact between LDs and mitochondria in these highly oxidative cells.

This review summarizes the evidence supporting the contact between LDs and mitochondria using morphological, biochemical, and biophysical data and focuses on interactions which cannot be separated by ultracentrifugation. This review brings forward a new point of view, proposing a distinction between stable and dynamic contact which can describe and explain some recent experimental findings. We propose that the stable interaction between LDs and mitochondria is maintained by rivet-like protein structures that prevent the separation between them during ultracentrifugation.

## Identification of Membrane Trafficking Proteins on Lipid Droplets

The potential trafficking capability of LDs was revealed through the visualization of LDs moving along microtubules, mediated by the protein Klar (Welte et al., 1998) and other motor proteins (Xie et al., 2019). Proteomic studies of isolated LDs identified Rab and SNARE proteins (Fujimoto et al., 2004; Liu et al., 2004). The targeting of trafficking proteins to LDs has been studied through fluorescent fusion proteins and immunogold-labeled electron microscopy, providing further evidence that LDs are involved in membrane trafficking (Martin et al., 2005; Liu et al., 2007). In functional studies LD-associated Rabs have been demonstrated to regulate interactions between LDs and ER (Rab 18) (Martin et al., 2005; Ozeki et al., 2005), LDs and mitochondria (Rabs and SNARE) (Jägerström et al., 2009; Pu et al., 2011), LDs and early endosomes (Rabs) (Liu et al., 2007), and to drive LD fusion (Rab8) (Wu et al., 2014). The movement of LDs throughout the cell with regulated interactions with other organelles hints at an active and important role for the LD in cellular lipid homeostasis.

The discovery of hormone-sensitive lipase (HSL) and PLIN1 on LDs, and the identification of the mechanism of activation and LD targeting of HSL via phosphorylation of both proteins, identified the catalytic function of this organelle. Additional proteomic studies found lipid synthetic enzymes on isolated LDs, pointing to an anabolic role (Fujimoto et al., 2004; Liu et al., 2004). In addition, some non-coding RNA-coded proteins are recently identified in isolated LDs (Huang et al., 2020). Functional studies of isolated LDs using *in vitro* assays confirmed that LD is a site for cellular lipid synthesis (Fujimoto et al., 2007; Zhang et al., 2016).

Thus, the LD is a site of neutral lipid storage, but is also metabolically active, can move rapidly throughout the cell, and possesses the molecular machinery to regulate interactions with other organelles. Collectively, these observations point to a central role for LDs in cellular lipid homeostasis.

## The Morphological Studies of Contact Between Lipid Droplets and Mitochondria

The interaction between LDs and mitochondria was first observed more than a half-century ago. George E. Palade was one of the major contributors who visualized the physical contact between LDs and mitochondria in guinea pig pancreas using transmission electron microscopy (TEM) (Figure 1Aa). EM also revealed the contact between these two organelles in mammary gland cells (Stemberger et al., 1984) and brown adipose tissue (BAT) (Figure 1Ab). Since these discoveries, many biologists have contributed to this field, accumulated visual evidence documenting the interactions between LDs and mitochondria by EM (Peute et al., 1978; Pu et al., 2011; Yu et al., 2015; Cui et al., 2019).

Advances in biological dyes, such as JC-1 and MitoTracker for mitochondria and Nile red, Bodipy, Oil Red O, and LipidTOX for LDs, have permitted the application of fluorescence microscopy to the study of LD-mitochondrion interactions (Figures 1Ac,d). The discovery of LD marker proteins enabled the use of immunolabeling and fluorescent fusion proteins. Together these advances have greatly simplified the study of the organellar interactions. Furthermore, the recent development of high resolution light microscopy techniques has facilitated finer observations, significantly aiding functional studies of the interactions between LDs and mitochondria (Valm et al., 2017).

Morphological studies have described highly dynamic interactions between LDs and mitochondria. For example, it was observed by EM that endurance exercise training enhances the physical contact between LDs and mitochondria in muscle cells. This finding is fully consistent with the upregulation of FA β-oxidation in mitochondria to meet the energetic needs during exercise (Tarnopolsky et al., 2007). In another study using live-imaging light microscopy, the distance between LDs and mitochondria was found to change in response to various experimental manipulations with close co-localization interpreted as direct contact (Valm et al., 2017).

A wide range of terminology has been applied to direct the contact between LDs and mitochondria. These include, but are not limited to, approximation, association, interaction, targeting, tethering, contact, binding, and anchoring. In one instance, the interaction site was called recognition site (Zehmer et al., 2009). Table 1 summarizes the most common terms used to describe inter-organelle interaction. Mainly based on morphologic observations, the current well-accepted term for the interaction between organelles, including LDs and mitochondria, is “contact” and the site for the interaction is termed as the “contact site.” Contact is characterized by: (1) co-localization of organelles, (2) the distance between organelles approximately 10–70 nm, (3) a contact site composed of tethering proteins, and (4) the ability to transfer ions, lipids, and other molecules (Schuldiner and Bohnert, 2017).

**TABLE 1:** Patterns of interaction between LDs and mitochondria.

Interaction terms	Organisms/tissues and cells	Methods	References
Interaction	Rat/skeletal muscle, L6	EM, LM, BpM, BcM, *In vitro* reconstitution	Pu et al., 2011
	Mouse/heart muscle	EM	Wang et al., 2013
	Monkey/kidney fibroblast cell	LM	Pribasnig et al., 2018
	Vero cell	EM	Herms et al., 2015
	Mouse/BAT	EM, LM, BpM, BcM	Yu et al., 2015
	*Phaeodactylum tricornutum*	EM	Lupette et al., 2019
Interaction (SNAP23)	NIH 3T3 cells	LM, BpM, BcM	Jägerström et al., 2009
Interaction (PLIN5)	CHO-K1 cell; AML12 cell; HL1 cell	BpM, BcM	Wang et al., 2011
	Human/skeletal muscle	EM, LM	Bosma et al., 2012; Gemmink et al., 2018
Interaction/contact (MFN2)	Human/BeWo cells	LM	Wasilewski et al., 2012
Interaction (RAB32)	Yellow catfish/hepatocytes	LM, BpM, BcM	Song et al., 2020
Interaction/association (MIGA2)	3T3-L1, COS7 cells	LM, EM, BpM, BcM	Freyre et al., 2019
Association	Mouse/BAT	LM	Benador et al., 2018
	Mouse/skeletal muscle, C2C12 cell	EM, LM, BpM, BcM	Zhang et al., 2011
	Mouse/Hepatocyte	EM	Shiozaki et al., 2011
	CHO Cell, 3T3-L1 fibroblasts	EM, LM	Murphy et al., 2009
Contact	Vero cell	EM	Barbosa et al., 2015
	MEF	LM	Rambold et al., 2015
	Dog/muscle cell (triceps)	EM	Vock et al., 1996
	Zebrafish/hepatocyte	EM	Peute et al., 1978
	Mouse/liver	EM	Krahmer et al., 2018
	Turtle/leydig cell	EM	Tarique et al., 2019
Contact (MFN2/PLIN1)	Mouse/BAT	EM, BpM, BcM	Boutant et al., 2017
Co-localization	Porcine/oocytes	LM	Sturmey et al., 2006; Milakovic et al., 2015
Adhere	Rat/mammary	EM	Stemberger et al., 1984
Close proximity	MEF	LM	Nguyen et al., 2017
Junction (PLIN5)	Mouse/cardiac tissue	EM	Varghese et al., 2019
Anchoring	Mouse/BAT	EM, LM, BpM, BcM	Cui et al., 2019

*LM, light microscopy; EM, electron microscopy; BpM, biophysical methods; BcM, Biochemical methods.*

The contact between LDs and mitochondria has been studied and visualized in many types of cells using a variety of technologies. However, molecular techniques are required to push our understanding further. As in other fields of biology, the powerful omics tools have been applied with great success. The first interactomic study screened LD and mitochondrial surface proteins, searching for interacting protein pairs using a bimolecular fluorescence complementation assay in *Saccharomyces cerevisiae* (Pu et al., 2011). In the same work an *in vitro* assay to study the interaction between isolated LDs and mitochondria was established, in which GTP was found to play a key role for this interaction (Pu et al., 2011). The most recent organelle-level interactomic study using high resolution microscopy visualized not only the interaction between LDs and mitochondria but also four other membranous organelles using a multispectral image acquisition method (Valm et al., 2017). Interactomic studies of this type provide a broad, system wide view of the network for interactions. However, insight into the mechanisms enabling these interactions requires other experimental techniques.

## The Biochemical and Biophysical Studies of Contact Between Lipid Droplets and Mitochondria

Biochemical and biophysical studies have been used to probe the molecular basis of inter-organelle contact. Contact has been dissected into discrete phases: recognition/targeting, tethering, and binding/anchoring, with distinct proteins playing key roles in each step. While the Rab proteins previously described are key candidates for recognition and tethering, other proteins including PLIN1, PLIN5, MFN2, and MIGA2 have also been implicated in these phases (Wasilewski et al., 2012; Boutant et al., 2017; Freyre et al., 2019; Varghese et al., 2019). Some proteins serve multiple functions. For example, VPS13C functions both as a binding component and plays a role in lipid transport (Kumar et al., 2018). Some proteins involved in LD-mitochondrion contact have been found to mediate the contact between other organelles as well. Studies using live-cell imaging have shown that these proteins are involved in highly dynamic contact, fitting a “kiss-and-run” model. Beyond morphologic observation, experiments using co-isolation (Figures 1Ba,b) and *in vitro* reconstitution have been used to further dissect the mechanisms of this contact (Pu et al., 2011).

Distinct from other membrane-bound organelles, the LD uniquely possesses a density less than 1 g/cm^3^ due to the TAG forming its core. This enables the method of floatation in a centrifugal field for the isolation/purification of LDs (Ding et al., 2013). All other membrane-bound organelles with densities higher than 1 g/cm^3^ are driven in the opposite direction into a pellet below the aqueous phase media. Therefore, a simple centrifugation can easily separate LDs from other membrane-bound organelles. Strong binding between LDs and other organelles could result in co-migration in the centrifugal field, either to the low-density region or the pellet.

In fact, other membranous organelles, such as ER and mitochondria, are commonly co-isolated with LDs and this co-fractionation historically was considered as contamination. Indeed, some membranous structures can be stripped from LDs through ultracentrifugation, but other structures remain tightly bound, suggesting that these co-fractionated organelles are bound physiologically in a type of organelle complex. The analysis of isolated and re-isolated LD fractions by EM and light microscopy provides visual evidence of intact membranous organelles bound to the surface of LDs, demonstrating the physiological and physical binding of LDs with other membranous organelles. The strength of the centrifugal field can be used to measure the binding strength, distinguishing weak contact (association/kiss-run) from strong contact (stable binding/anchoring). Therefore, this experimental approach can be used to classify types of contact.

This property of LDs can also be studied using *in vitro* assays (Pu et al., 2011). After incubation with controlled components, LDs can be re-isolated by floatation. Membranous structures induced to bind to the LDs in the *in vitro* system are co-floated. Utilizing this unique property of LDs, isolated LDs were incubated with isolated early endosomes in the presence or absence of GTP, the reaction mixture was centrifuged, and the re-isolated LDs were analyzed. Through this experiment, the physiological binding of early endosomes to LDs was identified to be regulated by GTP. Stripping Rab proteins from both isolated LDs and early endosomes totally blocked the binding, demonstrating that Rab protein(s) are key players governing the physiological binding between early endosomes and LDs (Liu et al., 2007).

A similar experiment was conducted to investigate factors that govern the binding between LDs and mitochondria. The two organelles, isolated separately, were mixed and incubated in the presence or absence of GTP or cytosol, followed by the re-isolation of the LDs. Mitochondria were co-fractionated with the floating LD fraction under the GTP condition, identifying GTP as a key factor regulating the binding between these two organelles (Pu et al., 2011).

Many mitochondrial proteins were identified in LDs isolated from mouse skeletal muscle using ultracentrifugation, indicating the strong contact between these two organelles (Zhang et al., 2011). Another study of LDs isolated from rat heart with ultracentrifugation also found numerous mitochondrial proteins (Li et al., 2016). A summary of finding from proteomic studies of isolated LDs shows that mitochondrial proteins co-fractionate with LDs isolated from a broad range of cell types (Table 2). Most of these studies used ultracentrifugation conditions, demonstrating the tight contact between these two organelles. It is also quite interesting that this phenomenon primarily is present in cells from highly oxidative tissues (Cui et al., 2019). The LD-mitochondrion contact is especially easy to visualize in BAT, an observation that is consistent with the role of these cells in heat production and the resulting high energy demand of mitochondria (Géloën et al., 1990; Ohue and Makita, 1992). Therefore, brown adipocytes represent a uniquely suitable experimental system for these investigations.

**TABLE 2:** Mitochondrial proteins from previously published LD proteomes.

Model/cell line	Total proteins	Mitochondrial (%)	LD isolation	References
Mouse/skeletal muscle	324	29.63	300,000 *g* for 60 min at 4°C	Zhang et al., 2011
Rat/heart	752	31.38	256,000 *g* for 60 min at 4°C	Li et al., 2016
Mouse/BAT	169	66.87	2,000 *g* for 3 min at 4°C	Yu et al., 2015
A431	32	9.38	274,000 *g* for 60 min at 4°C	Kim et al., 2006
CHO K2	125	16.8	274,000 *g* for 60 min at 4°C	Bartz et al., 2007b
3T3-L1	39	17.95	26,000 *g* for 30 min at 4°C	Brasaemle et al., 2004

Recent studies of isolated BAT LDs have suggested a distinction between two types of contact (Cui et al., 2019). The first is tight/stable contact (Cui et al., 2019), also called anchoring since it cannot be separated by ultracentrifugation, similar to the results from isolated LDs of muscle and heart (Zhang et al., 2011; Li et al., 2016). The other is dynamic contact that can be separated using high speed centrifugation (9,000*g*). The term peridroplet mitochondria (PDM) was proposed to describe these loosely associated mitochondria (Benador et al., 2018). After centrifugation with 9,000*g*, about 50% of LD-associated were still remained on re-isolated LD fraction. In addition, PDM are functionally different with cytosolic mitochondria and more toward to fatty acid synthesis (Benador et al., 2018). These differences can be understood physiologically due to the different demands placed on oxidative systems of the cell, depending on the role of the tissue.

## Dynamic and Stable Contacts Between Lipid Droplets and Mitochondria

The empirical distinction between weak and strong contact revealed by centrifugal force is likely to reflect important physiological complexity. The contact likely functions to permit efficient signaling and transport of hydrophobic molecules. For oxidative tissues such as heart muscle, skeletal muscle, and BAT, rapid delivery of FAs from LDs to mitochondria is an essential element for efficient ATP and heat production. The contact between these two organelles not only makes this delivery fast but also avoids insolubility and cytotoxicity of free FAs. Stable/anchoring contact found in these tissues (Cui et al., 2019) well represents the necessity for the constant production of ATP or heat with high efficiency (Figure 3). The term of LD-anchored mitochondria (LDAM) has been proposed for the mitochondria that stably associate with LDs (Cui et al., 2019). Both LDAM and PDM co-fractionate with LDs that are isolated with the low centrifugal force while only LDAM remain in the following centrifugation at speeds generating over 9,000*g*.

The current definition of contact is mainly based on morphological studies and includes four key characteristics that describe the dynamic contact well but seem to lack a criterion that defines the strength of contact. The nature of the molecules bridging these two organelles may explain the different strengths of contacts. Identifying the proteins mediating the different types of contact is important to understand the underlying physiology. Since LDAM are not affected in PLIN1 or PLIN5 deleted mouse BAT (Cui et al., 2019), the interaction between PLIN1 and MFN2 may be a good example of the protein pair involved in dynamic contact (Boutant et al., 2017; Olzmann and Carvalho, 2019). The proteins forming cellular tight junctions and the proteins with a nail (Havaux, 1998) or rivet-like (Iacovache et al., 2006) conformation may provide a useful model for understanding the stable contact (Figure 3). It is reasonable to include a fifth criterion classifying the contact based on its stability in a centrifugal field.

**FIGURE 2:**
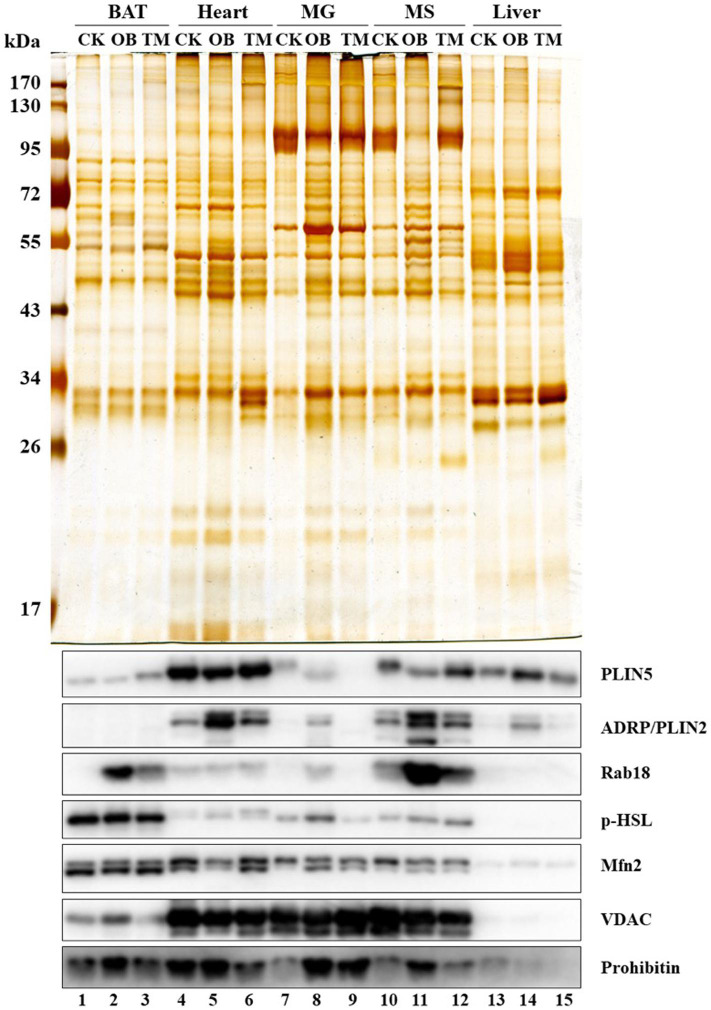
Co-isolation of LDs and mitochondria. Rhesus monkeys (*Macaca mulatta*) including Normal (CK), Obese (OB), and Type 2 diabetes mellitus (TM) were obtained from Kunming Institute of Zoology, Chinese Academy of Sciences. The LDs from different tissues of these monkeys were isolated using indicated homogenization methods and centrifugal forces as following: (1) A dounce type glass-teflon homogenizer and a speed of 247,000*g* were used for heart, musculus gastrocnemius (MG), and musculus soleus (MS). (2) A dounce type loose-fitting potter-elvehjem tissue grinder and a speed of 247,000*g* were used for liver. (3) A 200-mesh screen with plastic syringe piston and speeds of 1,000*g* and 247,000*g* were used for BAT. The proteins from isolated LDs were separated by SDS-PAGE and visualized by silver staining (upper panel). The marker proteins for LDs (PLIN5, ADRP/PLIN2, Rab18, and p-HSL) and for mitochondria (Mfn2, VDAC, and Prohibitin) were determined by Western blots (lower panel) (cite from Cui et al., 2019).

The LDAM have also been identified in the oxidative tissues of Rhesus monkeys (*Macaca mulatta*), including BAT (BAT), heart (Heart), and muscles (MG and MS) (Figure 2, lanes 1–12), but not in liver (Liver) (Figure 2, lanes 13–15), suggesting that the LDAM are conserved from mice to monkeys. To further understand the physiological significance of this stable contact (or LDAM), the monkeys with different metabolic conditions, such as normal (CK), obese (OB), and diabetes (TM), were utilized and the significant differences are found not only in LD-associated proteins but also in LDAM proteins (Figure 2).

**FIGURE 3:**
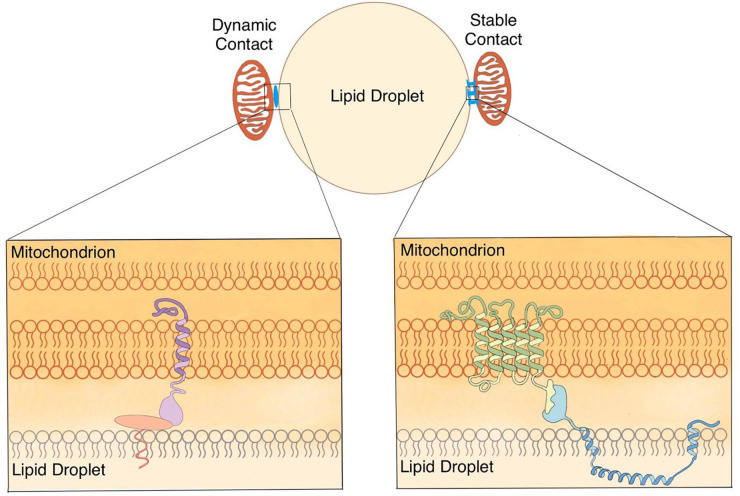
Anchoring hypothesis of the contact between LDs and mitochondria. Based on previous studies from ours and other laboratories, the working hypothesis is proposed that there are two types of contact (dynamic and stable) between LDs and mitochondria in oxidative cells. Dynamic contact can tether LDs and mitochondria through protein complexes. Stable contact can anchor mitochondria on LDs by nail- or rivet-like proteins.

Another fundamental question remaining to be answered for this anchoring hypothesis (Figure 3), is whether two types of contact are interchangeable based on physiological requirements. If this is possible, dynamic contact could transition to stable contact and back as energetic requirements fluctuate. Alternatively, if these are permanent states of association, there may be other important structural and functional differences between these two states. It is possible that mitochondria and LDs locked in stable contact coordinate in a manner similar to some protein complexes. In this case, it may be useful to think of LD and mitochondrion engaged in a stable contact as a distinct cellular structure, an organelle complex. Nevertheless, we suggest that both morphological and biochemical methods should be used together to study the contact between LDs and mitochondria as well as other organelle contacts.

To conduct the biochemical and biophysical study for the contact between LDs and mitochondria using isolation method, two critical factors should be addressed for isolation of LDs from BAT and brown adipocytes since they contain supersize LDs that are fragile and easy to be broken. These factors are (1) stringency of homogenization and (2) centrifugal force of floatation. Three homogenization methods that have been applied on BAT and brown adipocytes are listed by stringency (low to high): Nitrogen Cavitation technology (N_2_ bomb) (Cui et al., 2019), grinding tissue on 200-mesh screen using plastic syringe piston (Yu et al., 2015; Cui et al., 2019), and dounce homogenization (Benador et al., 2018). The centrifugal force has been utilized for sample from BAT and brown adipocytes ranging from 500 to 100,000*g* depending on the processes of isolation, washing, and separation. The high homogenization stringency and high centrifugal force can remove nonspecific bound contaminations but result missing of supersize LDs. But low centrifugal force cannot float small LDs efficiently (Zhang et al., 2016). Therefore, homogenization methods and centrifugal forces should be selected properly to control the quality, the size, and the type of LDs isolated from different tissues and cells.

Finally, based on previous studies from ours and other laboratories, the working hypothesis is proposed that there are two types of contact (dynamic and stable) between LDs and mitochondria in oxidative cells (Figure 3). The co-isolation method is introduced to study the contact between LDs and mitochondria, as well as other bilayer membrane-bound organelles. Combined with morphological and biochemical approaches, the co-isolation study is going to uncover new aspects toward to better dissect and understand the contact between these two organelles.

## Author Contributions

PL conceived the project. LC carried out the experiments and data analysis. LC and PL wrote the manuscript. Both authors contributed to the article and approved the submitted version.

## Conflict of Interest

The authors declare that the research was conducted in the absence of any commercial or financial relationships that could be construed as a potential conflict of interest.

## References

[B1] Barbosa A. D. Savage D. B. Siniossoglou S. (2015). Lipid droplet–organelle interactions: emerging roles in lipid metabolism. *Curr. Opin. Cell Biol.* 35 91–97. 10.1016/j.ceb.2015.04.017 25988547

[B2] Bartz R. Li W.-H. Venables B. Zehmer J. K. Roth M. R. Welti R. (2007a). Lipidomics reveals that adiposomes store ether lipids and mediate phospholipid traffic. *J. Lipid Res.* 48 837–847. 10.1194/jlr.m600413-jlr200 17210984

[B3] Bartz R. Zehmer J. K. Zhu M. Chen Y. Serrero G. Zhao Y. Liu P. (2007b). Dynamic Activity of Lipid Droplets: Protein Phosphorylation and GTP-Mediated Protein Translocation. *J. Proteome. Res.* 6, 3256–3265. 10.1021/pr070158j 17608402

[B4] Benador I. Y. Veliova M. Mahdaviani K. Petcherski A. Wikstrom J. D. Assali E. A. (2018). Mitochondria bound to lipid droplets have unique bioenergetics, composition, and dynamics that support lipid droplet expansion. *Cell Metab.* 27 869.e6–885.e6. 29617645 10.1016/j.cmet.2018.03.003 PMC5969538

[B5] Binns D. Januszewski T. Chen Y. Hill J. Markin V. S. Zhao Y. (2006). An intimate collaboration between peroxisomes and lipid bodies. *J. Cell Biol.* 173 719–731. 10.1083/jcb.200511125 16735577 PMC2063889

[B6] Bosma M. Minnaard R. Sparks L. M. Schaart G. Losen M. De Baets M. H. (2012). The lipid droplet coat protein perilipin 5 also localizes to muscle mitochondria. *Histochem. Cell Biol.* 137 205–216. 10.1007/s00418-011-0888-x 22127648 PMC3262136

[B7] Boutant M. Kulkarni S. S. Joffraud M. Ratajczak J. Valera-Alberni M. Combe R. (2017). Mfn2 is critical for brown adipose tissue thermogenic function. *EMBO J.* 36 1543–1558. 10.15252/embj.201694914 28348166 PMC5452040

[B8] Brasaemle D. Barber T. Wolins N. E. Serrero G. Blanchette-Mackie E. J. Londos C. (1997). Adipose differentiation-related protein is an ubiquitously expressed lipid storage droplet-associated protein. *J. Lipid Res.* 38 2249–2263. 9392423

[B9] Brasaemle D. L. Dolios G. Shapiro L. Wang R. (2004). Proteomic analysis of proteins associated with lipid droplets of basal and lipolytically stimulated 3T3-L1 adipocytes. *J. Biol. Chem.* 279 46835–46842. 10.1074/jbc.m409340200 15337753

[B10] Cui L. Mirza A. H. Zhang S. Liang B. Liu P. (2019). Lipid droplets and mitochondria are anchored during brown adipocyte differentiation. *Protein Cell* 10 921–926. 10.1007/s13238-019-00661-1 31667701 PMC6881423

[B11] Ding Y. Zhang S. Yang L. Na H. Zhang P. Zhang H. (2013). Isolating lipid droplets from multiple species. *Nat. Protoc.* 8:43. 10.1038/nprot.2012.142 23222457

[B12] Freyre C. A. C. Rauher P. C. Ejsing C. S. Klemm R. W. (2019). MIGA2 links mitochondria, the ER, and lipid droplets and promotes de novo Lipogenesis in Adipocytes. *Mol. Cell.* 76 811.e4–825.e4. 31628041 10.1016/j.molcel.2019.09.011

[B13] Fujimoto T. Ohsaki Y. Cheng J. Suzuki M. Shinohara Y. (2008). Lipid droplets: a classic organelle with new outfits. *Histochem. Cell Biol.* 130 263–279. 10.1007/s00418-008-0449-0 18546013 PMC2491702

[B14] Fujimoto Y. Itabe H. Kinoshita T. Homma K. J. Onoduka J. Mori M. (2007). Involvement of ACSL in local synthesis of neutral lipids in cytoplasmic lipid droplets in human hepatocyte HuH7. *J. Lipid Res.* 48 1280–1292. 10.1194/jlr.m700050-jlr200 17379924

[B15] Fujimoto Y. Itabe H. Sakai J. Makita M. Noda J. Mori M. (2004). Identification of major proteins in the lipid droplet-enriched fraction isolated from the human hepatocyte cell line HuH7. *Biochim. Biophys. Acta* 1644 47–59. 10.1016/j.bbamcr.2003.10.018 14741744

[B16] Géloën A. Collet A. Guay G. Bukowiecki L. J. (1990). In vivo differentiation of brown adipocytes in adult mice: an electron microscopic study. *Am. J. Anat.* 188 366–372. 10.1002/aja.1001880404 2392993

[B17] Gemmink A. Daemen S. Kuijpers H. J. H. Schaart G. Duimel H. Lopez-Iglesias C. (2018). Super-resolution microscopy localizes perilipin 5 at lipid droplet-mitochondria interaction sites and at lipid droplets juxtaposing to perilipin 2. *Biochim. Biophys. Acta Mol. Cell Biol. Lipids* 1863 1423–1432. 10.1016/j.bbalip.2018.08.016 30591149

[B18] Greenberg A. S. Coleman R. A. Kraemer F. B. Mcmanaman J. L. Obin M. S. Puri V. (2011). The role of lipid droplets in metabolic disease in rodents and humans. *J. Clin. Investig.* 121 2102–2110. 10.1172/jci46069 21633178 PMC3104768

[B19] Greenberg A. S. Egan J. J. Wek S. A. Garty N. B. Blanchette-Mackie E. Londos C. (1991). Perilipin, a major hormonally regulated adipocyte-specific phosphoprotein associated with the periphery of lipid storage droplets. *J. Biol. Chem.* 266 11341–11346. 2040638

[B20] Havaux M. (1998). Carotenoids as membrane stabilizers in chloroplasts. *Trends Plant Sci.* 3 147–151. 10.1016/s1360-1385(98)01200-x

[B21] Herms A. Bosch M. Reddy B. J. N. Schieber N. L. Fajardo A. Ruperez C. (2015). AMPK activation promotes lipid droplet dispersion on detyrosinated microtubules to increase mitochondrial fatty acid oxidation. *Nat. Commun.* 6:7176. 10.1038/ncomms8176 26013497 PMC4446796

[B22] Huang T. Bamigbade A. T. Xu S. Deng Y. Xie K. Ogunsade O. O. (2020). Identification of noncoding RNA-encoded proteins on lipid droplets. *Sci. Bull.* 10.1016/j.scib.2020.09.022 [Epub ahead of print]. 36654408

[B23] Iacovache I. Paumard P. Scheib H. Lesieur C. Sakai N. Matile S. (2006). A rivet model for channel formation by aerolysin-like pore-forming toxins. *EMBO J.* 25 457–466. 10.1038/sj.emboj.7600959 16424900 PMC1383540

[B24] Jägerström S. Polesie S. Wickström Y. Johansson B. R. Schröder H. D. Højlund K. (2009). Lipid droplets interact with mitochondria using SNAP23. *Cell Biol. Int.* 33 934–940. 10.1016/j.cellbi.2009.06.011 19524684

[B25] Jiang H.-P. Serrero G. (1992). Isolation and characterization of a full-length cDNA coding for an adipose differentiation-related protein. *Proc. Natl. Acad. Sci. U.S.A.* 89 7856–7860. 10.1073/pnas.89.17.7856 1518805 PMC49813

[B26] Kim S. C. Chen Y. Mirza S. Xu Y. Lee J. Liu P. (2006). A clean, more efficient method for in-solution digestion of protein mixtures without detergent or urea. *J. Proteome Res.* 5 3446–3452. 10.1021/pr0603396 17137347

[B27] Krahmer N. Najafi B. Schueder F. Quagliarini F. Steger M. Seitz S. (2018). Organellar proteomics and phospho-proteomics reveal subcellular reorganization in diet-induced hepatic steatosis. *Dev. Cell* 47 205–221.e207. 30352176 10.1016/j.devcel.2018.09.017

[B28] Kumar N. Leonzino M. Hancock-Cerutti W. Horenkamp F. A. Li P. Lees J. A. (2018). VPS13A and VPS13C are lipid transport proteins differentially localized at ER contact sites. *J. Cell. Biol.* 217 3625–3639. 10.1083/jcb.201807019 30093493 PMC6168267

[B29] Li L. Zhang H. Wang W. Hong Y. Wang J. Zhang S. (2016). Comparative proteomics reveals abnormal binding of ATGL and dysferlin on lipid droplets from pressure overload-induced dysfunctional rat hearts. *Sci. Rep.* 6:19782. 10.1038/srep19782 PMC4726412 26795240

[B30] Liu P. Bartz R. Zehmer J. K. Ying Y.-S. Zhu M. Serrero G. (2007). Rab-regulated interaction of early endosomes with lipid droplets. *Biochim. Biophys. Acta Mol. Cell Res.* 1773 784–793. 10.1016/j.bbamcr.2007.02.004 17395284 PMC2676670

[B31] Liu P. Ying Y. Zhao Y. Mundy D. I. Zhu M. Anderson R. G. (2004). Chinese hamster ovary K2 cell lipid droplets appear to be metabolic organelles involved in membrane traffic. *J. Biol. Chem.* 279 3787–3792. 10.1074/jbc.m311945200 14597625

[B32] Lupette J. Jaussaud A. Seddiki K. Morabito C. Brugiere S. Schaller H. (2019). The architecture of lipid droplets in the diatom *Phaeodactylum tricornutum*. *Algal Res.* 38:101415 10.1016/j.algal.2019.101415

[B33] Martin S. Driessen K. Nixon S. J. Zerial M. Parton R. G. (2005). Regulated localization of Rab18 to lipid droplets: effects of lipolytic stimulation and inhibition of lipid droplet catabolism. *J. Biol. Chem.* 280 42325–42335. 10.1074/jbc.m506651200 16207721

[B34] Milakovic I. Jeseta M. Hanulakova S. Knitlova D. Hanzalova K. Hulinska P. (2015). Energy status characteristics of porcine oocytes during *in vitro* maturation is influenced by their meiotic competence. *Reprod. Domest Anim.* 50 812–819. 10.1111/rda.12592 26280917

[B35] Murphy D. J. (2001). The biogenesis and functions of lipid bodies in animals, plants and microorganisms. *Prog. Lipid Res.* 40 325–438. 10.1016/s0163-7827(01)00013-3 11470496

[B36] Murphy D. J. (2012). The dynamic roles of intracellular lipid droplets: from archaea to mammals. *Protoplasma* 249 541–585. 10.1007/s00709-011-0329-7 22002710

[B37] Murphy S. Martin S. Parton R. G. (2009). Lipid droplet-organelle interactions; sharing the fats. *Biochim. Biophys. Acta Mol. Cell Biol. Lipids* 1791 441–447. 10.1016/j.bbalip.2008.07.004 18708159

[B38] Nguyen T. B. Louie S. M. Daniele J. R. Tran Q. Dillin A. Zoncu R. (2017). DGAT1-dependent lipid droplet biogenesis protects mitochondrial function during starvation-induced autophagy. *Dev. Cell* 42 9–21.e25. 10.1016/Fj.devcel.2017.06.003 28697336 PMC5553613

[B39] Ogasawara Y. Tsuji T. Fujimoto T. (2020). Multifarious roles of lipid droplets in autophagy – Target, product, and what else?. *Semin. Cell Dev. Biol.* [Epub ahead of print]. 10.1016/j.semcdb.2020.02.013 32169402

[B40] Ohue M. Makita T. (1992). Interrelationship between lipid droplets and mitochondria in brown adipocytes of the hamster. *J. Vet. Med. Sci.* 54 1131–1135. 10.1292/jvms.54.1131 1477163

[B41] Olzmann J. A. Carvalho P. (2019). Dynamics and functions of lipid droplets. *Nat. Rev. Mol. Cell Biol.* 20 137–155. 10.1038/s41580-018-0085-z 30523332 PMC6746329

[B42] Ozeki S. Cheng J. Tauchi-Sato K. Hatano N. Taniguchi H. Fujimoto T. (2005). Rab18 localizes to lipid droplets and induces their close apposition to the endoplasmic reticulum-derived membrane. *J. Cell Sci.* 118 2601–2611. 10.1242/jcs.02401 15914536

[B43] Peute J. Van Der Gaag M. A. Lambert J. G. D. (1978). Ultrastructure and lipid content of the liver of the zebrafish, Brachydanio rerio, related to vitellogenin synthesis. *Cell Tissue Res.* 186 297–308. 10.1007/bf00225539 627021

[B44] Pribasnig M. Kien B. Pusch L. Haemmerle G. Zimmermann R. Wolinski H. (2018). Extendedresolution imaging of the interaction of lipid droplets and mitochondria. *Biochim. Biophys. Acta Mol. Cell Biol. Lipids* 1863 1285–1296. 30305245 10.1016/j.bbalip.2018.07.008

[B45] Pu J. Ha C. W. Zhang S. Jung J. P. Huh W.-K. Liu P. (2011). Interactomic study on interaction between lipid droplets and mitochondria. *Protein Cell* 2 487–496. 10.1007/s13238-011-1061-y 21748599 PMC4875178

[B46] Rambold A. S. Cohen S. Lippincott-Schwartz J. (2015). Fatty acid trafficking in starved cells: regulation by lipid droplet lipolysis, autophagy, and mitochondrial fusion dynamics. *Dev. Cell* 32 678–692. 10.1016/j.devcel.2015.01.029 25752962 PMC4375018

[B47] Ramos S. V. Macpherson R. E. Turnbull P. C. Bott K. N. Leblanc P. Ward W. E. (2014). Higher PLIN5 but not PLIN3 content in isolated skeletal muscle mitochondria following acute in vivo contraction in rat hindlimb. *Physiol. Rep.* 2:e12154. 10.14814/phy2.12154 25318747 PMC4254090

[B48] Roberts M. A. Olzmann J. A. (2020). Protein quality control and lipid droplet metabolism. *Annu. Rev. Cell Dev.Biol.* 36 115–139. 10.1146/annurev-cellbio-031320-101827 33021827 PMC7593838

[B49] Schuldiner M. Bohnert M. (2017). A different kind of love – lipid droplet contact sites. *Biochim. Biophys. Acta Mol. Cell Biol. Lipids* 1862 1188–1196. 10.1016/j.bbalip.2017.06.005 28627434

[B50] Shiozaki M. Hayakawa N. Shibata M. Koike M. Uchiyama Y. Gotow T. (2011). Closer association of mitochondria with lipid droplets in hepatocytes and activation of Kupffer cells in resveratrol-treated senescence-accelerated mice. *Histochem. Cell Biol.* 136 475–489. 21818579 10.1007/s00418-011-0847-6

[B51] Shulman G. I. (2014). Ectopic fat in insulin resistance, dyslipidemia, and cardiometabolic disease. *N. Engl. J. Med.* 371 1131–1141. 10.1056/nejmra1011035 25229917

[B52] Stemberger B. H. Walsh R. M. Patton S. (1984). Morphometric evaluation of lipid droplet associations with secretory vesicles, mitochondria and other components in the lactating cell. *Cell Tissue Res.* 236 471–475. 6733773 10.1007/BF00214252

[B53] Song Y.-F. Hogstrand C. Ling S.-C. Chen G.-H. Luo Z. (2020). Creb-Pgc1α pathway modulates the interaction between lipid droplets and mitochondria and influences high fat diet-induced changes of lipid metabolism in the liver and isolated hepatocytes of yellow catfish. *J. Nutr. Biochem.* 80:108364. 10.1016/j.jnutbio.2020.108364 32199344

[B54] Sturmey R. G. O’toole P. J. Leese H. J. (2006). Fluorescence resonance energy transfer analysis of mitochondrial:lipid association in the porcine oocyte. *Reproduction* 132 829–837. 10.1530/rep-06-0073 17127743

[B55] Tarique I. Vistro W. A. Bai X. Yang P. Hong C. Huang Y. (2019). LIPOPHAGY: a novel form of steroidogenic activity within the LEYDIG cell during the reproductive cycle of turtle. *Reprod. Biol. Endocrinol.* 17:19. 10.1186/s12958-019-0462-2 30738428 PMC6368689

[B56] Tarnopolsky M. A. Rennie C. D. Robertshaw H. A. Fedak-Tarnopolsky S. N. Devries M. C. Hamadeh M. J. (2007). Influence of endurance exercise training and sex on intramyocellular lipid and mitochondrial ultrastructure, substrate use, and mitochondrial enzyme activity. *Am. J. Physiol. Regul. Integr. Comp. Physiol.* 292 R1271–R1278. 17095651 10.1152/ajpregu.00472.2006

[B57] Tauchi-Sato K. Ozeki S. Houjou T. Taguchi R. Fujimoto T. (2002). The surface of lipid droplets is a phospholipid monolayer with a unique fatty acid composition. *J. Biol. Chem.* 277 44507–44512. 10.1074/jbc.m207712200 12221100

[B58] Thiam A. R. Farese R. V. Jr. Walther T. C. (2013). The biophysics and cell biology of lipid droplets. *Nat. Rev. Mol. Cell Biol.* 14 775–786. 10.1038/nrm3699 24220094 PMC4526153

[B59] Valm A. M. Cohen S. Legant W. R. Melunis J. Hershberg U. Wait E. (2017). Applying systems-level spectral imaging and analysis to reveal the organelle interactome. *Nature* 546:162. 10.1038/nature22369 28538724 PMC5536967

[B60] Varghese M. Kimler V. A. Ghazi F. R. Rathore G. K. Perkins G. A. Ellisman M. H. (2019). Adipocyte lipolysis affects Perilipin 5 and cristae organization at the cardiac lipid droplet-mitochondrial interface. *Sci. Rep.* 9:4734. 10.1038/s41598-019-41329-4 PMC6426865 30894648

[B61] Vock R. Hoppeler H. Claassen H. Wu D. X. Billeter R. Weber J. M. (1996). Design of the oxygen and substrate pathways. VI. structural basis of intracellular substrate supply to mitochondria in muscle cells. *J. Exp. Biol.* 199:1689. 10.1242/jeb.199.8.1689 8708576

[B62] Walther T. C. Farese R. V. (2012). Lipid droplets and cellular lipid metabolism. *Annu. Rev. Biochem.* 81 687–714. 10.1146/annurev-biochem-061009-102430 22524315 PMC3767414

[B63] Wang H. Lei M. Hsia R.-C. Sztalryd C. (2013). “Chapter 8–analysis of lipid droplets in cardiac muscle,” in *Methods in Cell Biology*, eds Yang H. Li P. (Cambridge, MA: Academic Press), 129–149. 10.1016/b978-0-12-408051-5.00008-5 PMC7446730 24099291

[B64] Wang H. Sreenivasan U. Hu H. Saladino A. Polster B. M. Lund L. M. (2011). Perilipin 5, a lipid droplet-associated protein, provides physical and metabolic linkage to mitochondria. *J. Lipid Res.* 52 2159–2168. 10.1194/jlr.m017939 21885430 PMC3220284

[B65] Wasilewski M. Semenzato M. Rafelski S. M. Robbins J. Bakardjiev A. I Scorrano L. (2012). Optic atrophy 1-dependent mitochondrial remodeling controls steroidogenesis in trophoblasts. *Curr. Biol.* 22 1228–1234. 10.1016/j.cub.2012.04.054 22658590 PMC3396839

[B66] Welte M. A. Gross S. P. Postner M. Block S. M. Wieschaus E. F. (1998). Developmental regulation of vesicle transport in *Drosophila* embryos: forces and kinetics. *Cell* 92 547–557. 10.1016/s0092-8674(00)80947-2 9491895

[B67] Wu C. C. Howell K. E. Neville M. C. Yates J. R. III Mcmanaman J. L. (2000). Proteomics reveal a link between the endoplasmic reticulum and lipid secretory mechanisms in mammary epithelial cells. *Electrophoresis Int. J.* 21 3470–3482. 10.1002/1522-2683(20001001)21:16<3470::aid-elps3470>3.0.co;2-g 11079566

[B68] Wu L. Xu D. Zhou L. Xie B. Yu L. Yang H. (2014). Rab8a-AS160-MSS4 regulatory circuit controls lipid droplet fusion and growth. *Dev. Cell* 30 378–393. 10.1016/j.devcel.2014.07.005 25158853

[B69] Xie K. Zhang P. Na H. Liu Y. Zhang H. Liu P. (2019). MDT-28/PLIN-1 mediates lipid droplet-microtubule interaction via DLC-1 in *Caenorhabditis elegans*. *Sci. Rep.* 9:14902. 10.1038/s41598-019-51399-z PMC6797801 31624276

[B70] Xu S. Zhang X. Liu P. (2018). Lipid droplet proteins and metabolic diseases. *Biochim. Biophys. Acta Mol. Basis Dis.* 1864 1968–1983. 10.1016/j.bbadis.2017.07.019 28739173

[B71] Yu J. Zhang S. Cui L. Wang W. Na H. Zhu X. (2015). Lipid droplet remodeling and interaction with mitochondria in mouse brown adipose tissue during cold treatment. *Biochim. Biophys. Acta* 1853 918–928. 10.1016/j.bbamcr.2015.01.020 25655664

[B72] Zechner R. Madeo F. Kratky D. (2017). Cytosolic lipolysis and lipophagy: two sides of the same coin. *Nat. Rev. Mol. Cell Biol.* 18:671. 10.1038/nrm.2017.76 28852221

[B73] Zehmer J. K. Huang Y. Peng G. Pu J. Anderson R. G. W. Liu P. (2009). A role for lipid droplets in inter-membrane lipid traffic. *Proteomics* 9 914–921. 10.1002/pmic.200800584 19160396 PMC2676673

[B74] Zhang C. Liu P. (2019). The new face of the lipid droplet: lipid droplet proteins. *Proteomics* 19:1700223. 10.1002/pmic.201700223 30216670

[B75] Zhang H. Wang Y. Li J. Yu J. Pu J. Li L. (2011). Proteome of skeletal muscle lipid droplet reveals association with mitochondria and apolipoprotein a-I. *J. Proteome Res.* 10 4757–4768. 10.1021/pr200553c 21870882

[B76] Zhang S. Wang Y. Cui L. Deng Y. Xu S. Yu J. (2016). Morphologically and functionally distinct lipid droplet subpopulations. *Sci. Rep.* 6:29539. 10.1038/srep29539 PMC4937419 27386790

